# Choroidal Metastasis Revealing a Lung Adenocarcinoma: A Case Report

**DOI:** 10.7759/cureus.18968

**Published:** 2021-10-22

**Authors:** Narjisse Aichouni, Hamid Ziani, Imane Kamaoui, Siham Nasri, Imane Skiker

**Affiliations:** 1 Radiology, Mohammed VI University Hospital, Oujda, MAR; 2 Faculty of Medicine and Pharmacy, Mohammed First University, Oujda, MAR

**Keywords:** imaging, adenocarcinoma, cancer, lung, choroidal metastasis

## Abstract

Choroidal metastasis is the most common malignant intraocular tumor. Its diagnosis in a patient with known lung cancer is usually easy. However, without any context of already known cancer, further elements are needed to guide the diagnosis. We report the case of a 47-year-old patient with a history of smoking who presented a choroidal metastasis of left lower pulmonary lobe adenocarcinoma discovered on imaging. Imaging techniques, mainly ultrasonography, CT scan, and MRI can help guide the diagnosis of choroidal metastasis even in the absence of a known origin initially.

## Introduction

The lung constitutes approximately 30% of choroid metastases. The incidence of metastasis of lung carcinomas into choroid is 2%-6.7% [1]. The symptoms of vision impairment help in the discovery of these tumours. The diagnosis of ocular lesions requires using different imaging techniques, mainly ultrasonography, computed tomography (CT) scan, and magnetic resonance imaging (MRI). This case report highlights that choroidal metastasis without a known primary should be supplemented with systemic as well as imaging studies.

## Case presentation

**Figure 1:**
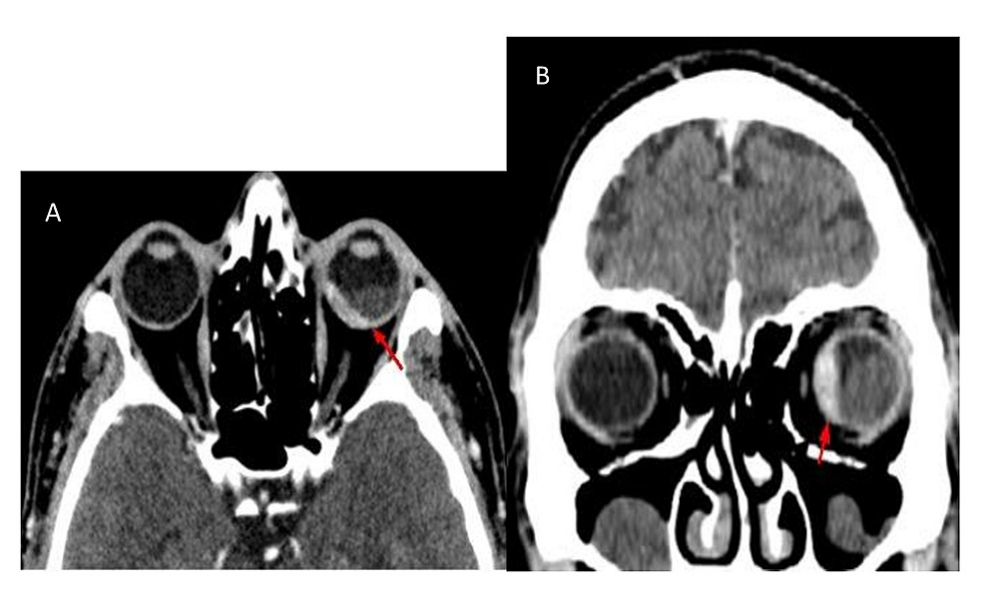
Axial (A) and coronal (B) enhanced CT images showing choroidal thickening of the left eye.

**Figure 2:**
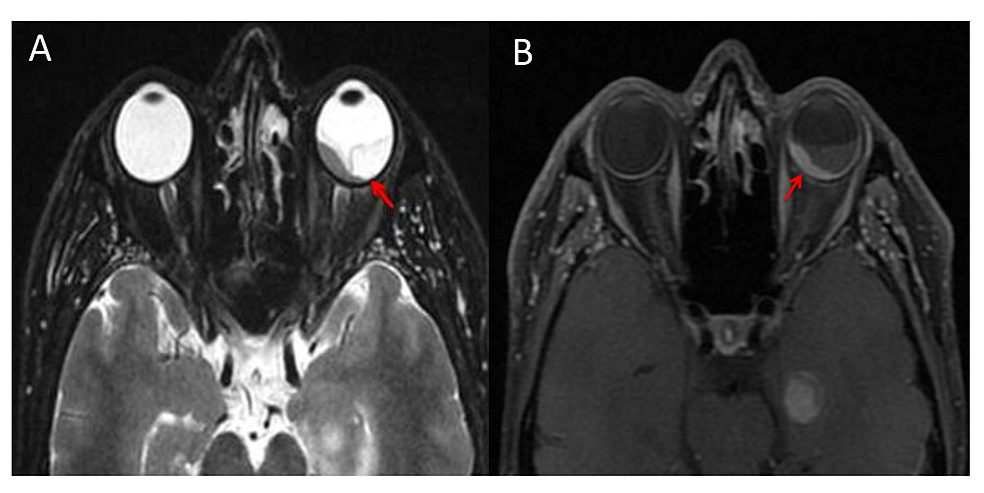
Axial T2 (A) and T1 weighted fat saturation after Gadolinium administration. (B) Magnetic resonance images showing choroidal lesion and retinal detachment of the left eye.

**Figure 3:**
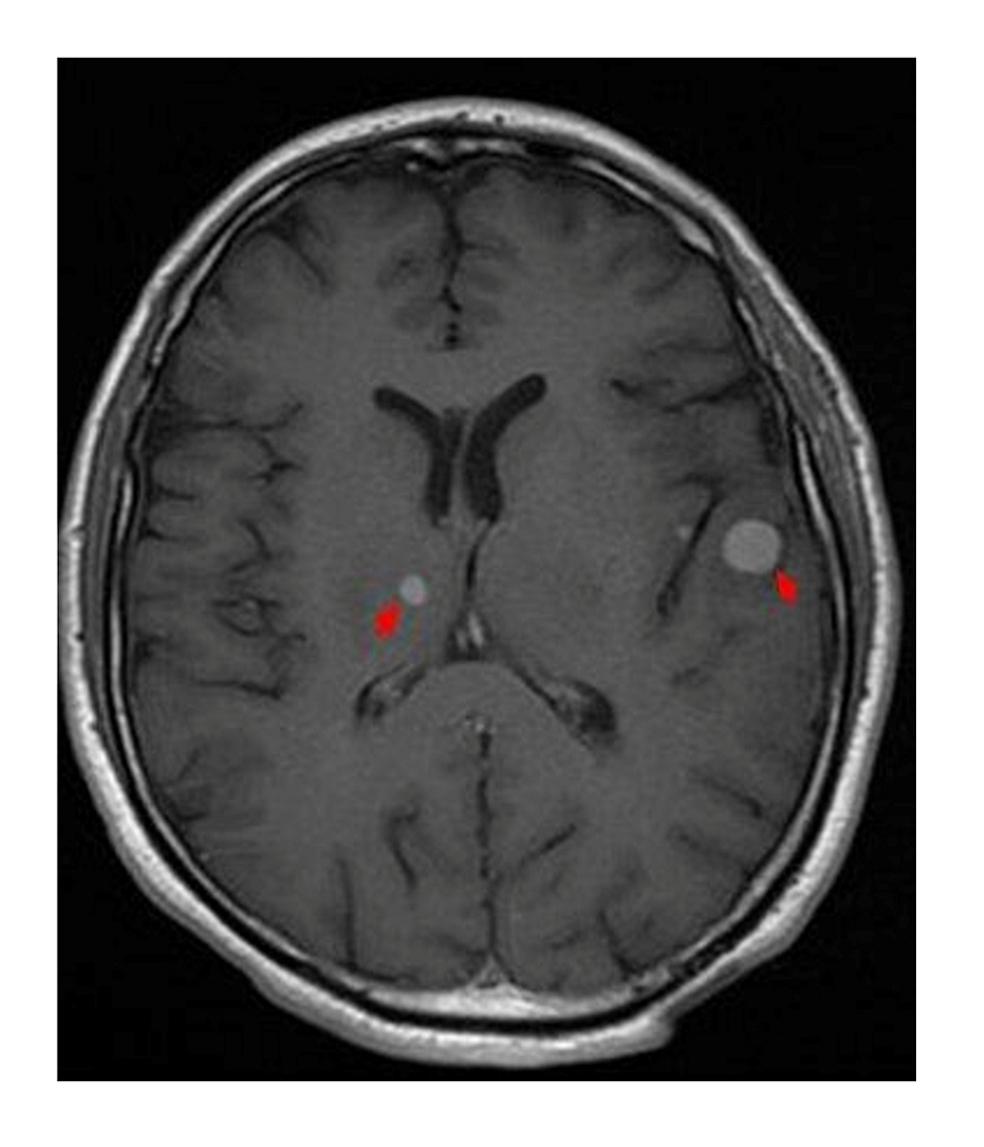
Axial T1 contrast gadolinium-enhanced image of brain secondary lesions

**Figure 4:**
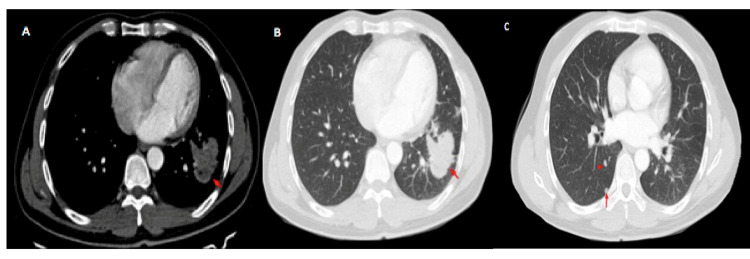
Axial CT images of left lower lobe lung tumoral process (A, B) and contralateral tumor nodules (C).

A 47-year-old man with a history of smoking was admitted for exploration of a dry chronic cough with decreased visual acuity in his left eye. Ocular examination revealed retinal detachment in the left eye. The right eye was normal. Ocular ultrasound showed a uveal juxtapapillary echogenic process responsible for retinal detachment which was consistent with enhanced choroidal thickening on the CT scan (Figure 1). MRI showed an intraocular lesion of the posterior wall of the posterior chamber, isointense on T1, discretely hyperintense on T2, enhanced after injection of gadolinium, with a retinal detachment (Figure 2). Brain MRI showed multiple nodular intra-axial brain secondary lesions hypointense on T1, hyperintense on T2 FLAIR, and enhancing intensely after injection of gadolinium (Figure 3). The diagnosis of choroidal metastasis was suspected, and given the clinical context, a chest CT was performed, which showed a tumoral process of the lower lobe of the left lung with contralateral tumor nodules (Figure 4). The bronchoscopic biopsy of the left lower lobe mass was taken and the pathological examination showed a poorly differentiated adenocarcinoma. The patient was referred to oncology for palliative radiotherapy followed by combination chemotherapy.

## Discussion

The incidence of intraocular metastases is about 8-10% [2]. After breast cancer, lung tumors are the second most important origin of CM representing 20 to 29 % of cases [3]. Adenocarcinoma is the most frequently found histological type followed by squamous cell carcinoma and small cell carcinoma [4]. Decreased visual acuity, phosphenes, floaters, and ocular pain are symptoms by which CM may be revealed, but incidental discovery is also possible [3]. Ophthalmological examination shows a subretinal mass of variable characteristics according to the nature of the original tumor, but yellow subretinal mass with subretinal fluid is the most common aspect [5]. CM may be the mode of discovery of an unknown lung tumor. In this case, diagnosis is more difficult and imaging has a major role for orientation and elimination of differential diagnosis, mainly CM.

On ophthalmoscopic examination, CM often have overlying subretinal fluid and lipofuscin that typically appear as scattered clumps of brown pigment. Autofluorescence shows hypoautofluorescence of the tumor with overlying areas of bright 3+ hyperautofluorescence correlating to the deposits of lipofuscin and 2+ hyperautofluorescence of subretinal fluid [6].

Ultrasonography shows flat or slightly curved lesions, often multilobular, with an irregular surface, with a heterogeneous echogenicity (medium-high), and often associated with serous retinal detachment, unlike choroidal melanomas which are rather homogeneous and moderately echogenic [7]. In our case, the lesion was echogenic with a retinal detachment.

MRI is more sensitive and specific than CT in the detection of intraocular tumors. MRI often shows a well-demarcated choroidal mass that appears isointense on T1 weighted images and hypointense on T2 weighted sequences [4]. In our case, the lesion was isointense on T1, discretely hyperintense on T2, and enhanced after injection of gadolinium with a retinal detachment.

The treatment of choroidal metastasis depends on the systemic status, number of choroidal tumors, location, and laterality. Ocular radiotherapy is considered a standard treatment option for intraocular metastatic tumors [2]. Systemic chemotherapy, immunotherapy, hormone therapy, or whole eye radiotherapy are performed if the metastases are multifocal and bilateral. Plaque radiotherapy, transpupillary radiotherapy, or photodynamic therapy are suggested for solitary metastasis, and enucleation for blind painful eyes [6]. Survival of lung cancer patients following the discovery of CM is 12 months [3].

## Conclusions

The lung is the second most common primary site of choroidal metastasis. The diagnosis of CM can be difficult if the primary tumor is unknown, and imaging has a major role in the orientation of the diagnosis and the search for primary cancer through CT scan and MRI.
